# Extraordinary Adaptive Plasticity of Colorado Potato Beetle: “Ten-Striped Spearman” in the Era of Biotechnological Warfare

**DOI:** 10.3390/ijms17091538

**Published:** 2016-09-13

**Authors:** Aleksandar Cingel, Jelena Savić, Jelica Lazarević, Tatjana Ćosić, Martin Raspor, Ann Smigocki, Slavica Ninković

**Affiliations:** 1 Plant Physiology Department, Institute for Biological Research “Siniša Stanković”, University of Belgrade, Bulevar despota Stefana 142, 11060 Belgrade, Serbia; cingel@ibiss.bg.ac.rs (A.C.); tatjana@ibiss.bg.ac.rs (T.Ć.); martin@ibiss.bg.ac.rs (M.R.); slavica@ibiss.bg.ac.rs (S.N.); 2 Insect Physiology and Biochemistry Department, Institute for Biological Research “Siniša Stanković”, University of Belgrade, Bulevar despota Stefana 142, 11060 Belgrade, Serbia; jellaz@ibiss.bg.ac.rs; 3 Molecular Plant Pathology Laboratory, USDA-ARS, 10300 Baltimore Avenue, Beltsville, MD 20705, USA; ann.smigocki@ars.usda.gov

**Keywords:** Colorado potato beetle, plant-insect co-evolution, biological control, transgenic potato

## Abstract

Expanding from remote areas of Mexico to a worldwide scale, the ten-striped insect, the Colorado potato beetle (CPB, *Leptinotarsa decemlineata* Say), has risen from being an innocuous beetle to a prominent global pest. A diverse life cycle, phenotypic plasticity, adaptation to adverse conditions, and capability to detoxify or tolerate toxins make this insect appear to be virtually “indestructible”. With increasing advances in molecular biology, tools of biotechnological warfare were deployed to combat CPB. In the last three decades, genetically modified potato has created a new challenge for the beetle. After reviewing hundreds of scientific papers dealing with CPB control, it became clear that even biotechnological means of control, if used alone, would not defeat the Colorado potato beetle. This control measure once again appears to be provoking the potato beetle to exhibit its remarkable adaptability. Nonetheless, the potential for adaptation to these techniques has increased our knowledge of this pest and thus opened possibilities for devising more sustainable CPB management programs.

## 1. Introduction

Within the last two centuries, Colorado potato beetle (CPB, *Leptinotarsa decemlineata* Say, Coleoptera: Chrysomelidae) has risen from being classified as a specialist to a relative generalist, and from a harmless beetle gained ranking of a leading global pest. CPB has originated in central Mexico where it fed on only a few wild hosts. Nowadays, the beetle is found throughout the Northern hemisphere and has adapted to about 20, both wild and cultivated solanaceous plants, with most severe attacks still occurring on its preferred host, the cultivated potato, *Solanum tuberosum* L. Defoliation by this voracious pest can cause 40%–80% yield losses in potato crops [1] and even total yield loss may occur if defoliation intensity reaches 75% [2].

Therefore, many different agricultural practices have been employed during the long history in combat with CPB, such as crop rotation, cultivating away from previous potato fields, putting up physical barriers, thermal or electromagnetic control (microwaves radiation), the insect removal using machinery (e.g., Beetle Eater^®^, Bio-Collector^®^, Bug-Buster^®^) [3], but the main control strategy still relies on the use of chemical pesticides. Long history of relatively unsuccessful physical and chemical CPB control measures, representing “125 years of mismanagement” [4], opened the space for implementation of biological means of control as more pest specific and less risky for the environment. The so-called second green revolution that occurred at the beginning of the 1980s has led to the proposed usage of biorational insecticides and antifeedants, natural enemies, trap cropping and genetic modification of plants for CPB control.

Introduction of recombinant proteins with insecticidal properties into potato has been recognized as a promising approach for CPB control. After about three decades of intensive research, we review the effectiveness of this approach and other novel techniques that could surmount the noted problems and limitations.

## 2. From Innocuous Beetle to Superpest

During the mid-1860s, the CPB colonized potato in the Midwest United States and spread further by speed of 100 km per year [5] reaching the US West Coast in about 20 years later. In the early 20th century, the CPB was accidentally introduced in France. Based on low genetic variability of European CPB populations as compared to North American, it has possibly been a single but very successful invasion on Europe [6]. With an average invasion speed of 50 km per year [5], the beetle has conquered most of Central Europe in about 30 years [6]. Today, it continues its northerly spread towards Scandinavia and Siberia. With an imposing eastward speed of 150 km per year [7], at the end of the 20th century, CPB expanded through parts of Asia Minor, Central Asia and China, threatening colonization of other suitable regions that include India, Korea, Japan, parts of Africa, South America, New Zealand and Australia [8].

**Figure 1:**
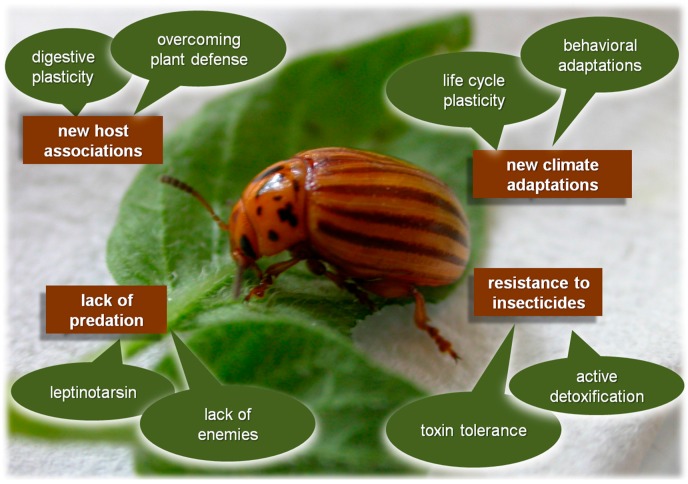
Different eco-physiological adaptations that give the Colorado potato beetle (CPB) characteristics of one of the most invasive species worldwide, and the “superpest” status.

CPB invasion from Mexican highlands to the current worldwide distribution actually results from evolution of various eco-physiological adaptations (Figure 1). Primarily, success of CPB outspreading has been based on the evolution of new insect-plant interactions. Since “old” hosts are still used along with “new” ones, this process should be referred to host expansion, rather than host shift [9]. For example, CPB populations from Mexico show significantly greater preference and performance on its ancestral weedy host *S. rostratum*, than on its novel host *S. tuberosum.* On the other hand, as a result of adaptation of North American beetle populations to cultivated potato, equal preference and performance have been recorded on both hosts [10]. Current CPB populations also show diversity of new host associations, and in some regions, CPB evolved improved fitness on other cultivated solanaceous plants becoming the primary pest on eggplant (*S. melongena*) and tomato (*S. lycopersicum*). In addition, host range of derived CPB populations is not limited to cultivated *Solanaceae*. This beetle also utilizes some of the wild *Solanum* plants such as nightshades (e.g., *S. dulcamara* and *S. elaeagnifolium*) or nettles (e.g., *S. carolinense*), and although fitness traits are worse on weedy species compared to cultivated potato [11], the alternative hosts may provide additional food resources in seasons before development of potato leaves or when potato is exhausted during CPB outbreak.

Moreover, CPB populations possess enough genetic variation in life history traits that is a prerequisite for the evolution of adaptive strategies in new environments. Following the expansion of potato cultivation areas, CPB evolved adaptations for various adverse conditions. For instance, compared to the other European CPB populations, Russian populations that are exposed to adverse low temperatures, exhibit faster development, higher survival rate, tendency to grow larger in size [12], and bury deeper in the soil for overwintering [13], probably due to faster food consumption or more efficient food utilization.

CPB is a very successful conqueror for several additional reasons. First, both CPB larva and adult produce leptinotarsin, a neurotoxin which protects them from predation. Some arthropod parasitoids and predators such as lady beetles (*Coleomegilla maculata*), stink bugs (*Perillus bioculatus*) or ground beetles (*Lebia grandis*) can, however, significantly reduce CPB populations [14,15,16]. However, density of these natural enemies of CPB in potato fields is usually not sufficient to endanger numerous CPB populations, and not all of predators can follow the CPB to newly colonized areas. For instance, from the 1960s, there were attempts to introduce an American CPB predator, *P. bioculatus*, in Europe, but despite its mass release in potato fields in Slovakia, France, Germany, Poland, Russia, Italy and Hungary, this predator has not become naturalized anywhere in Europe [17].

Second, CPB is famous for rapid build-up of resistance to insecticides. Although hundreds of compounds have been tested against it, CPB remains one of the main challengers of the modern pesticide industry. Whatever was used against it—arsenicals, organochlorines, carbamates, organophosphates or pyrethroids—could not break the adaptive machinery of CPB. Induction of detoxifying enzymes, reduced insecticide penetration, increased insecticide excretion or target site mutation that render insecticide insensitivity [8] are some of the known mechanisms of CPB resistance associated with more than 50 different active ingredients in nearly all insecticide classes [18]. Due to rapid evolution of CPB resistance, more chemical insecticides are applied to control this pest which has to cope with at least 0.6 t of various insecticide active compounds used annually in developed countries [19]. It is impressive to note that some of the newly applied insecticides failed during the second, or even during the first year [20]. Even imidacloprid, a neonicotinoid effectively used for almost 10 years, was starting to fail at the beginning of this century [21,22]. Although many new insecticides are developed and tested against CPB [23], there is no ground for reliance that any of them will successfully manage this voracious beetle.

Finally, some aspects of the beetle life cycle and its plasticity bring many advantages to CPB. Depending on climate, the beetle can have several generations annually and the females are known to have high fecundity, with more than 3000 eggs per female in laboratory conditions [24]. In large populations, there is a high probability of existence of genetic variants that provide high fitness under new conditions, as well as appearance of new adaptive random mutations. Due to multivoltinism (i.e., several generations annually) these old and new genetic variants can be rapidly favored by natural selection and provide enlargement of adapted populations. In addition, until their reproductive maturation, about 25% of young adults stay close to the place of their larval and pupal development, and adapted individuals can produce homozygous resistant offsprings [25]. Most CPB females enter winter diapause fertile and a portion of the population may extend the diapause for two or more years. This trait can reduce efficacy of customized management strategies such as crop and insecticide rotations or, for instance, enable CPB to stay dormant and skip adverse years only to re-establish population when conditions become favorable.

## 3. Go behind Enemy Lines

**Figure 2:**
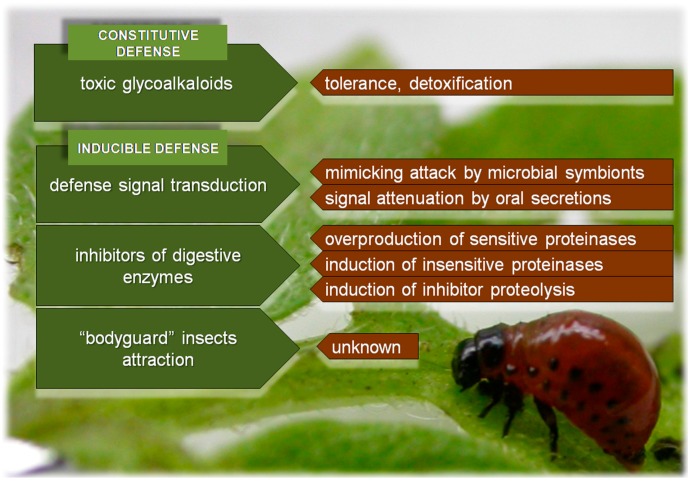
Potato defense strategies (green boxes) and the reciprocal CPB countermeasures (red boxes) that reflect co-evolutionary “arms race” between these two species.

Emerging as a co-evolutionary game that now has been playing for more than 400 million years [26], interactions between herbivorous insects and plants are fascinating in complexity and dynamics. The insects are continuously adapting to efficiently exploit their hosts while, at the same time, plants rapidly evolve new mechanisms to counteract the insect attackers [27]. In this biological warfare, plants and insects continuously go head to head in their interactions, changing each other. Particularly, CPB has evolved broad physiological adaptations to its hosts and some of the known aspects of co-evolutionary “arms race” between potato and CPB are presented in Figure 2. These traits can also serve as preadaptations that allowed CPB to become an “archetype” for resistance development to the man-made control measures [28].

Adult CPB is attracted to hosts by plant volatile organic compounds (VOCs) that induce positive anemotaxis. The beetle is able to perceive some of these volatiles in the field at a distance of up to 50 m from its source [29], and the sensitivity and attraction by the potato VOCs are increased with insect hunger and sexual maturation [30]. Hereafter, feeding decision is made by contact chemoreception of plant compounds that CPB recognizes as phagodeterrents and/or phagostimulants [31,32].

The CPB distinguishes potato from other plants by its ability to recognize the specific blend of potato VOCs, and very interestingly, only a whole set of potato VOCs in a very specific ratio can attract CPB [33]. CPB shows attraction to mature undamaged potato, but there is no attraction to younger plants [34]. However, when young potato plants are damaged, VOCs emission is up to ten-fold higher than in undamaged plants and positive anemotaxis is exhibited by CPB [34]. There are two stages of attraction induced by different sets of potato volatiles. First attraction is to the VOCs released directly by mechanical wounding that disappears about one hour after short term CPB feeding. Second attraction is to the different set of VOCs induced specifically by insect feeding in case of prolonged herbivory [34]. Such attractions are interesting because it is assumed that preference for undamaged plants represents an advanced adaptive strategy in order to avoid competition and induced plant defense. Moreover, some insects are repelled from further colonization by herbivory induced plant volatiles [35]. However, knowing that the male aggregation pheromone has been attractive to both male and female CPB and that significantly more adults are attracted to the potato already infested with male beetles [36], it seems that by using both pheromone and plant volatile signals, these beetles colonize potato for feeding and, more importantly, for reproduction.

As a part of constitutive defense against herbivory, solanaceous plants are loaded with glycoalkaloids (Figure 2), such as solanine, chaconine, leptines and demissine that act mainly as phagodeterrents, while some of them can have negative effects on CPB larval development [37]. However, CPB has been specialized for solanaceous plants due to its ability to tolerate [38] or detoxify host plant allelochemicals [39]. Moreover, CPB survival and growth have been unaffected by feeding on cultivated potato varieties that are selected against high glycoalkaloid level [40].

Therefore, potato has to rely on the next line of defense, the inducible one (Figure 2). Both mechanical wounding and the components of CPB regurgitant elicit potato defense responses. Plants can differentiate wounding from insect infestation [41], and the regurgitant-responsive genes include both genes that respond exclusively to insect feeding and wounding-responsive genes whose expression could be modulated by insect feeding. It has been shown that 73 potato genes were induced, while 54 genes were repressed by adding CPB regurgitant to wounded leaves [42]. In addition, CPB feeding modulated expression of 29 leaf proteins and 16 of them were down-regulated, whereas only 8 proteins change expression in response to wounding [43]. A number of expressed genes correspond to the stress-related inducible proteins that include proteinase inhibitors (PIs), pathogen response proteins, peroxidases and polyphenol oxidases, compounds that are important in direct plant defense. Moreover, expression of genes encoding compounds involved in indirect defense, such as volatiles that attract potato beetle natural enemies, is also elevated by CPB feeding.

Although CPB attack initiates the potato defense machinery, it is interesting to note that some of the genes are actually down-regulated by the feeding but nonetheless are included as part of the direct defense response. For instance, it has been shown that an isoform of potato aspartate PI, a wound inducible inhibitor of aspartic proteinases in CPB midguts, is downregulated by CPB oral secretions while the second isoform is slightly repressed despite its upregulation in wounded potato leaves [43]. In the same manner, expression of two serine PIs, PIN1 and PIN2 (wound-induced proteinase inhibitor 1 and 2), is repressed by CPB regurgitant in wounded tomato leaves [44]. Additionally, CPB regurgitant contains symbiotic bacteria that are secreted during feeding. These CPB microbial symbionts interfere in insect-plant interactions [45]. The host plant is also misled by the symbionts in perceiving the CPB attack as microbial thus mustering inadequate plant defense response against the attacking CPB. Namely, microbial infection initiates the salicylic acid signaling pathway for immune responses that in *Solanum* plants attenuate antiherbivory responses, mediated by jasmonic acid. Such suppression by which CPB circumvents host plant defense mechanisms undoubtedly brings an adaptive advantage for this insect. In contrast, downregulation of photosynthesis-related components, provoked by CPB feeding but not by mechanical wounding [43], corresponds to the potato adaptive response to the insect attack. This can represent both a metabolic strategy for relocation of carbon resources towards defense [46] and also lower nutritive value of plant leaves that can have a negative impact on insect fitness [47].

Despite the sabotage of defense signal cascades by CPB, high amounts of PIs are still synthesized in potato leaves in response to beetle attack [48]. However, in its combat arsenal, CPB possesses different strategies to overcome this line of potato defense. To encounter the high level of potato PIs, CPB increases production of “ordinary” proteolytic enzymes and induces new isoforms [48] and classes of digestive proteinases [49] that are insensitive to potato inhibitors. Additionally, among CPB proteinases induced or upregulated as a response to potato PIs are those that can cleave and thus inactivate the potato PIs [48]. As a result of these responses, CPB larvae adjust their digestive proteolysis to be unaffected by potato PIs (Figure 2). However, for such adaptation to overcome the negative effect of the inducible potato defense, CPB has to pay a metabolic price of about 30% reduction of potential fitness [50].

In addition, CPB oviposition on potato leaves is recognized by the plant that can react by forming necrosis around the laid eggs, a phenomenon first observed in Polish potato fields [51]. The foliar hypersensitive response is also documented on hybrid potato varieties where necrosis was followed by egg dislodgement from the leaves [52]. Although eggs displacement from leaves to soil has no effect on eclosion or on ability of newly hatched larvae to find potato, the percent of neonates that succeed in colonizing potato was almost zero under field conditions, likely due to increased predation by the ground beetles [52]. However, this rare but potentially very useful mechanism of host plant resistance is inconsistent among potato cultivars and it is not clear if its appearance may be the result of potato-CPB co-evolution [52].

## 4. One More in a Line

Insecticidal crystalline proteins from *Bacillus thuringiensis* (Bt; Cry toxins) are utilized for insect pest control as an alternative to chemical insecticides. Cloning, transfer and expression of Cry toxin genes in transgenic plants is generally adopted as a strategy for incorporating resistance in commercially important crops. In the US, for instance, Bt-cotton represents 73% of the total cotton production while Bt-corn accounts for 63% of all corn grown [53].

When insects feed on Bt-plants, ingested Cry protoxin is proteolytically activated in insect midguts to the active toxin. Binding of the active toxin to epithelial cell receptors causes midgut tissue disruption that leads to halt of insect feeding and subsequent larval mortality. Since the Cry toxins act like any other dietary protein in human gut for which epithelial cells lack appropriate receptors, they do not bear substantial risk for human health [54], and are also harmless to other vertebrates and majority of beneficial insects [55].

In 1995, when Monsanto released Cry3A-transgenic NewLeaf™, potato became one of the first commercialized Bt-modified crops. Although very effective in preventing CPB damage, reducing use of the chemical pesticides and increasing yield, NewLeaf™ was withdrawn from the markets in 2001 due to public concerns, and to date has never been returned to the fields. In potato, Cry toxin was expressed at a very high level relative to the CPB susceptibility. Toxin levels were at least 50 and 20 times higher that necessary to kill CPB first instar or for stopping adult feeding, respectively [56]. Although very effective, the high dose strategy represents extremely high selection pressure for developing resistance in the insect populations, and without additional management practices, it has been predicted that CPB can develop resistance to Bt-potato within at most 10 generations [57]. The potential for CPB developing resistance to Bt toxins has been demonstrated in the laboratory by repeated Cry3A toxin application [58], and relative to the susceptible beetles, selection resulted in about 300-fold increase in resistance ratio after 35 generation [59]. Moreover, resulting resistant CPB population was able to survive on Bt-potato [60].

In difference to sensitive strains, Bt-resistant CPB strain exhibits at least two levels of adaptive responses that render immunity to the Cry3A toxin [61]. First are the changes in digestive enzyme profiles and specific increase in aminopeptidase activity that, although are not connected with alteration in toxin processing or its inactivation, correlate with CPB Cry toxin resistance. It is known that proteinases in insect midguts are involved in modulation and amplification of signals that activate specific innate immune responses such as melanization, coagulation and defense peptide synthesis [62], mechanisms confirmed to be employed in overcoming the exposure to Bt toxin in other insect species [63,64]. The second level that contributes to CPB resistance is lower toxin binding to the receptors. Since the two CBP populations did not differ in binding affinity [61], lower toxin binding could be a consequence of a reduced number of binding sites within the receptor molecule or a reduced number of receptors. Such traits may lead to a heritable advantage that can allow CPB to survive on Bt-potato.

In addition, physiological resistance to Bt-potato, in both CPB larvae and adults, is reinforced by behavioral resistance. While the movement of susceptible beetles is arrested with Bt-potato leaf ingestion, the resistant CPB larvae tend to escape from the toxin and, interestingly, more resistant larvae show higher behavioral responsiveness [65]. In a similar way, ingestion of Bt toxin increases flight activity of resistant adult beetles [66]. Such behavioral differences between susceptible and resistant CPB can affect gene flow, increasing distribution of resistant homozygous offsprings within and between Bt-potato fields [60].

The development of new Bt-varieties is ongoing (i.e., Guo and collaborators [67] generated selectable marker-free transgenic Bt-potato), with expectations that Bt-potatoes could once again enter the market. However, for sustainability of the Bt approach, the most important concern is whether CPB can be prevented from developing resistance to Bt-potato? The proposed management strategies, that fit to the biology of CPB, include nontransgenic potato refuges along the transgenic potato fields, with no or very rational use of insecticides [68]. By reducing selection pressure, such strategies can prevent accumulation of rare homozygous resistance genes in Bt-exposed CPB population and, keep Bt-potato “in business” for longer time. However, since insect resistance is a process of evolution responding to selection pressure, it is hard to believe that CPB problem on potato can be permanently solved by the Bt approach alone. In 2013, almost two decades after first commercialization of Bt-crops, the most targeted pest populations still remain susceptible, but reduced efficacy of Bt-plants caused by field-evolved resistance has been reported in 5 of 13 major crop pest populations, compared to only one reported in 2005 [69].

## 5. Compensating for Loss

Targeting insect digestive proteinases as a strategy in plant protection arose after it was reported that soybean PIs had a negative effect on red flour beetle larvae [70]. The first PI-transgenic plant, tobacco expressing cowpea CpTI trypsin proteinase inhibitor [71], exhibited increased resistance to several targeted insect pests. After this initial success, numerous PI-transgenic plants showing elevated pest resistance were generated, but to date, none have been commercialized [72]. Although inhibition of digestive proteolysis by recombinant PIs leads to essential amino acid deficiency [73] and can result in target pest death, it rather causes a decrease in insect fitness components other than survival. However, slight reduction in insect growth and reproduction or prolonged larval development can negatively affect pest population dynamics and lead to large reduction in population size. For example, longer larval development means longer exposure to predators and pathogens, while the reduction in body mass can decrease the insect’s investment in reproduction. This approach also minimizes the possible development of insect resistance and thus represents an alternative control strategy to the “acute mortality” approach executed by Bt toxins and chemical insecticides. However, insect adaptive capacities in some cases compromise success of this control strategy, and as a reflection of long co-evolutionary process, insect can readily apply different physiological responses to target recombinant PIs. These responses commonly include overproduction of sensitive digestive enzymes that exceed the level of inhibitors, induction of different mechanistic classes or proteinase isoforms insensitive to the recombinant PI or that are capable of degrading the introduced PI [74,75].

For digestive proteolysis, CPB utilizes cysteine and aspartate proteinases [76], along with minor activity of serine and metalloproteinases [77]. Cathepsin B- and H-like cysteine proteinases make up to 66% of CPB digestive proteolysis, while cathepsin D-like aspartate proteinases contribute about 35% [78].

After encouraging results with E-64 [78], a specific broad spectrum cysteine PI from *Aspergillus japonicum*, CPB cysteine proteinases have become targets for heterologously expressed cystatins, a group of cysteine PIs. As the first potent candidates for challenging CPB, oryzacystatins I and II (OCI and OCII) isolated from rice and specifically active against cathepsin H, were emerged. Both PIs showed great potential in inhibition of CPB larval proteases in vitro [79], as well as in control of other Coleopteran pests [80,81]. However, CPB larvae overcome ingestion of rice cystatins in transgenic potato by hypertrophic behavior [82,83,84] and by inducing OCs insensitive isoforms that gradually restore inhibited cysteine proteinase activity [82,84]. It is fascinating to note that some aspects of larval performance have been actually improved by ingestion of recombinant OCs, i.e., growth and leaf consumption were faster, development period was shorter, and even final body mass was increased in case of OCI [82]. OCII, however, had slightly negative impact on larval mass [83], and such effect was more pronounced when both OCI and OCII inhibitors were simultaneously expressed in potato [84,85]. Further, when cathepsin B-like fraction of CPBs cysteine digestive proteolysis was challenged by a modified variant of cystatin from barley, CPB larvae chose another strategy to compensate for the loss of proteolytic activity. Namely, they hyperproduced sensitive proteinases [86].

Insects “recognize” PIs by specificity of enzyme-inhibitor interaction, translating them into transcriptional response that results in alteration in the digestive proteinase complement [87]. This implies that insect larvae would more “precisely” respond to more specific recombinant PI (i.e., by insensitive proteinase production), while the inhibitors with lower affinity for the insect proteinases would rather initiate hyperproduction of PI sensitive proteinases [88]. Additionally, induction of insensitive proteinases is a more successful adaptive response and, on an evolutionary time scale, it appeared after proteinase hyperproduction [89]. Good example of this appeared when CPB cathepsin D-like aspartate activity, important in initiation of protein hydrolysis [90], was targeted with recombinant cathepsin D inhibitor (CDI) from tomato. In a combat with CDI expressed in potato, CPB larvae initially responded with rapid overproduction of CDI sensitive proteinases that resulted in slight reduction of insect growth rate [91]. Hereafter, this first compensatory response to CDI was followed by a gradual decrease in CDI sensitive proteinase activity and a switch to alternative and insensitive proteinase complement which despite the presence of the CDI inhibitor enabled sustained larval growth and development [91].

The observed limited efficiency of recombinant PIs is not surprising since CPB is actually “overadapted” to survive the initial contact with host plant cysteine and aspartate PIs with about 60% of its proteolytic activity being sufficient to allow normal CPB growth and development [92]. This beetle is capable to easily broaden its digestive capacity when native inhibitors are additionally induced in potato or in less hostile plants such as tomato [92,93]. Furthermore, CPB possesses an extraordinary ability to specifically and rapidly adjust its proteolysis to the host PIs, exhibiting a variety of digestive compensatory responses to functionally different defense proteins [87,93,94,95]. Even in the case of deleterious E-64 ingestion, which covers the whole complement of CPB cysteine digestive proteinases, the beetle has retained the ability for some form of adaptation by introducing PI insensitive proteinases [78].

CPB larvae possess a wide variety of genes for digestive proteinase isoforms, expression of which can be modulated as a response to the induced host plant PIs. All of these isoforms have a common feature of substituted and/or inserted amino acids in the position near substrate binding sites [96]. Such small changes in amino acid sequences can provide strong steric hindrance in inhibitor interaction with the enzyme and differentiate between PI sensitive and insensitive CPB digestive proteinase isoforms. Furthermore, it is speculated that such minor structural modifications represent a common mechanism for advancement of digestive enzymes insensitive to plant PIs that can also be involved in insect’s adaptation to recombinant PIs [96,97].

In order to defeat the exceptional capability of CPB to make such compensations, broadening the spectrum of digestive inhibition, using transgene stacking, protein fusion or naturally present multidomain PIs, promised a fruitful strategy [98]. Use of a single and narrow spectrum CDI only slightly affected CPB larvae performance on transgenic potato [99]. However, application of a hybrid CDI-CCII inhibitor (fusion of CDI with corn cystatin II), active against different protease classes, reduced CPB growth and potato leaf consumption by about 50% [99]. The ability of CPB larvae to overcome inhibition of both aspartate and cysteine digestive proteinases remains to be tested by chronic ingestion of recombinant CDI-CCII.

Like the stacking of rice cystatins showing no stronger effect than the same inhibitors expressed individually [84], multidomain PIs from animal kingdom expressed in potato also appeared less promising for CPB control. Two domain serine PI from locust (*Schistocerca gregaria*) caused only a slight reduction in CPB larval performance [100,101]. The potent PI from the sea anemone (*Actinia equine*), equistatin, with activity against both cysteine and aspartyl proteinases in vitro had a somewhat unexpected fate in transgenic potato [102]. Although ingestion of equistatin-coated potato leaves caused almost complete retardation of CPB larval growth that was accompanied by a significant increase in mortality [103], its expression in transgenic potato failed to reduce CPB feeding since it was recognized as a foreign protein and cleaved by native potato proteinases thus destroying its activity [102].

Alternatively, expression of fungal cysteine PIs, macrocypin and clitocypin, in potato appeared more promising for CPB control [104,105]. These broad spectrum inhibitors of CPB cysteine proteinases reduced CPB larval growth and prolonged their development in a dose dependent manner [104,105]. The most interesting trait of macrocypin and clitocypin was the apparent lack of CPB digestive compensatory responses, which to date is characteristic for all tested PIs from other sources [104,105]. However, the current problem is their relatively low expression level achieved in transgenic potato for more convincing effects in CPB control.

All things considered, the use of a combination of novel and potent PIs is still deemed the most promising approach for augmenting the digestive proteinase inhibition spectrum for effective CPB control [87]. However, although the presence of the transgene cystatins in the human diet should not raise public concerns [106], expression of the strong aspartic and serine PIs in crops used as food or feed, could jeopardize this strategy and impose a need for development of alternatives in this approach to combat CPB.

## 6. Hopes for the Future?

At this time, there are no commercially available potato cultivars resistant to CPB, and it is highly unlikely that they will be available in the near future. However, despite the perceived dismal outlook, scientists have not run out of possible ammunition for combating CPB. Although it is questionable whether CPB can be stopped by resistance factors that exist in *Solanum* species [107], potato wild relatives are still considered a potential source of resistance traits. However, such trait introduction by traditional breeding is time consuming and suffers from difficulties such as crossing barriers and incorporation of undesirable traits. Genetic engineering can overcome these constraints and additionally provide for introduction of insecticidal traits derived from other sources that include the animal and microbial kingdoms. Further, despite the disappointing results due to the inherent complexity of CPB digestive proteases and its striking ability to compensate for the loss of digestive proteolytic activity, the PI approach should not yet be discarded as ineffective. When it comes to biological means of control, even a relatively small decrease in insect fitness can have a significant effect on population reduction, especially when it is used in combination with other control measures. Thus, a continued challenge for biotechnology could be to find or create variants of PIs that trigger minimal CPB compensation and are specific to CPB proteases without affect on non-target organisms. To further enhance the effectiveness of this approach, gene manipulation methods need to be improved and new ones developed in order to achieve more sustainable and “cleaner” technologies. Development of methods that enable precise plant genome editing using sequence-specific nucleases (SSN) could declare a new era on incorporating PIs or other insecticidal traits into potato [108]. However, despite the fact that gene transformation in potato is well established and that the potato genome sequence is now available [109], genome editing is still not commonly done in potato, and to the best of our knowledge, has not been reported for incorporation of CPB resistance. RNA interference (RNAi), a gene silencing mechanism mediated by double-stranded RNA (dsRNA), could also be a potential robust tool for CPB control on potato [110]. The main advantage of the RNAi approach is the availability of the CPB transcriptome [111] that allows for specific targeting of CPB genes critical for normal growth and development of the pest [112,113]. Just recently, the dsRNA strategy was employed to produce CPB resistant transgenic potato [114]. Targeting the CPB essential cytoskeletal protein β-actin caused 100% mortality of the beetle in 5 days. However, the major limitation of the RNAi based gene silencing approach is delivering and integrating transgenes in a random fashion. Most RNAi genome modifications were shown to be partial and not stable from one generation to the next.

But for sustainable CPB control, commonly used approaches need to be modified and improved. The ones relying on insecticides are inevitable, just like the evolution of CPB resistance to them. However, the rate at which resistance develops can vary with management strategies. For instance, existence of chemically untreated potato refuges reduces the selection pressure and can serve as a pool for susceptibility alleles that prevent fixation of resistance genes within CPB populations [115].

While less effective than insecticides, many other methods of CPB control are available. Integrated into scientifically sound management approach under the auspices of Integrated Pest Management (IPM) and adapted to the CPB biology, each of these strategies can augment the reduction of CPB population size and keep damage caused by this insect below the economical threshold. Adequately designed crop rotation or planting late potato cultivars can decrease CPB population buildup. For instance, even a seven-day delay in the colonization of potato fields in the spring can dramatically reduce the number of larvae late in the summer [116]. Knowledge of CPB chemical communication, although still incompletely understood, can be implemented in the strategies for potato beetle control. Disorientation of CPB adults by masking potato VOCs using intercrop cultures or antifeedants can reduce colonization of potato. As an example, CPB infestation can be decreased 60%–100% when tansy (*Tanacetum* *vulgare* L.) is intercropped in potato fields [117]. Furthermore, the use of trap crops with attractants and/or aggregation pheromone can significantly reduce the amount of insecticide needed for potato field treatment, while attractants coupled with chemical insecticides in trap crops can have similar control efficiency as insecticides, with application of only 8% of the insecticide active compound [118]. In addition, natural enemies of CPB can become a valuable part of management programs. Because of reciprocal adaptations it is not likely that CPB can develop resistance to their natural enemies. Using more selective biopesticides supported by adequate habitat design can increase the enemies’ impact on CPB population. For instance, mulching crop fields with wheat or rye straw creates a microenvironment that favors CPB predators, resulting in at least two-fold reduction in potato defoliation and significant increase in potato yield [119]. Thus, all of these “old-fashion” methods can be reinforced by biotechnology derived weapons against CPB, providing more sustainable and environmentally friendly control of potato beetle. Recent experiences with implementation of the simplest and basic IPM practices such as combining rationally used chemical insecticides with trap rows and crop rotation in some parts of the USA [28] proved its potential to make potato production more sustainable in the years to come.

Finally, to avoid the endless cycle of replacement of one failed measure with an effective new one that will fail in the future, there are two simple premises that we should keep in mind: (1) beside the fact that it is impossible, it is unnecessary to kill every potato beetle in the field since potato can tolerate defoliation to some extent without significant yield reduction and (2) the stronger the selection pressure is, especially if relying on a single control measure, the faster CPB can develop adaptive countermeasures, regardless of the approach used. However, developing efficient CPB countermeasures to the well suited and balanced “multiple attacks” approach suggested by IPM, would require complex adaptations that are highly unlikely to happen compared to the occurrence and fixation of random single gene mutations that can render insects insensitive to insecticides, Bt toxin or PIs.
